# Supplementary material for: No apparent trade-offs associated with heat tolerance in a reef-building coral
Source: Commun Biol. 2023 Apr 12;6:400. doi: 10.1038/s42003-023-04758-6 (PMC10097654; doi:10.1038/s42003-023-04758-6)
Supplement: Supplementary file 2 — Supplementary Material [file 42003_2023_4758_MOESM2_ESM.pdf]

No apparent trade-offs associated with heat tolerance in a reef-building coral

Liam Lachs[1*], Adriana Humanes[1], Daniel R. Pygas[2,3], John C. Bythell[1], Peter J. Mumby[4,5], Renata Ferrari[2], Will F. Figueira[3], Elizabeth Beauchamp[1], Holly K. East[6], Alasdair J. Edwards[1], Yimnang Golbuu[5], Helios M. Martinez[1], Brigitte Sommer[3,7], Eveline van der Steeg[1], James R. Guest[1]

[1]School of Natural and Environmental Sciences, Newcastle University, Newcastle upon Tyne, NE1 7RU, UK

[2]Australian Institute of Marine Sciences, Townsville, QLD 4810, Australia

[3]School of Life and Environmental Sciences, University of Sydney, Sydney, NSW 2006, Australia

[4]Marine Spatial Ecology Lab, School of Biological Sciences, University of Queensland, St. Lucia, QLD 4072, Australia

[5]Palau International Coral Reef Center, Koror 96940, Palau

[6]Department of Geography and Environmental Sciences, Northumbria University, Newcastle upon Tyne, UK

[7]School of Life Sciences, University of Technology Sydney, Sydney, NSW 2007, Australia

*Corresponding Author: Liam Lachs, l.lachs2@newcastle.ac.uk

Supplementary Materials

Supplementary Text 1: Characterisation of Symbiodiniaceae community composition

DNA from coral branch tips was extracted using the Qiagen DNeasy blood and tissue kit with overnight proteinase K digestion. Internal transcribed spacer 2 (ITS2) ribosomal marker genes (~450 bp) in Symbiodiniaceae algal symbionts, extraction blanks (without tissue), and PCR-negative controls (without DNA) were amplified via first-round PCR using the SYM_VAR_5.8S2 (5'-TCGTCGGCAGCGTCAGATGTGTATAAGAGACAGGA ATTGCAGAACTCCGTGAACC-3') and SYM_VAR_REV (5'-GTCTCGTGGGCTCGGAGATGTGTATAAGAGACAGCGGGTTCWCTTGTYTGACTTCATGC-3') primers following Voolstra et al. (2020) . Samples were then indexed using Illumina Nextera DNA unique dual indexes and underwent quality control using the Nanondrop One C assay and size assessment using the Tapestation 4200 D1000. The pooled equilibrated samples were sequenced as paired end reads on an Illumina MiSeq v3 with addition of 20% PhiX control' Cambridge, UK. Sequence data were analysed through Symportal [2], a pipeline designed for the analysis of Symbiodiniaceae ITS2 data. Based on all the ITS2 sequences within a sample Symportal determines complex ITS2 type profiles ('defining intragenomic variants', DIVs) that reflect the most abundant ITS2 sequences. DIVs reflect genetically distinct Symbiodiniaceae communities, where constituent members are ranked by abundance, including very low abundant ITS2 sequences that would have been below the detection limit of other methods, for example, the 97% operational taxonomic unit (OTU) approach [2]. DIVs constituents reflect different Symbiodiniaceae lineages (e.g., C40) as determined by numerous other genetic techniques.

Supplementary Text 2: 3D photogrammetry growth estimation extended methodology

The imaging and photogrammetry methods applied here followed [3], but can briefly be summarised by the following steps (Fig. S3). Refer to the methods of that study for further information.

Image acquisition. The imaging protocol for surveying each coral colony involved taking 200-300 images of the colony using a GoPro Hero action camera (v5 or 7) set to timelapse mode at 1 image per second (Fig. S3). An At least two calibration objects (Rubik's cubes in 2017 and coded targets from Agisoft Metashape [50mm diameter, 60mm apart] in 2018 and 2019) were placed as close to the colony as possible without obscuring the camera's view of the colony surface. Images were captured by moving the camera in an arch from the substrate to directly above the colony, taking 6-10 images along the way. This was repeated about 16 times moving radially around the colony. A further set of images was taken by moving the camera in two downward-facing spirals starting at the top of the colony and rotating in opposite directions down to the substrate next to the colony. Each pass captured a series of overlapping images with a minimum of 80% overlap.

3D colony models. 114 three-dimensional reconstructions were built across 45 colonies using Metashape Professional (v 1.5, Agisoft). Initial manual checks of images were done to remove blurry or irrelevant images and then the Image Quality Tool in Metashape was run to further identify blurry and low contrast images. Images with a quality of lower than 0.5 were generally excluded from further use in model building. Next, a standard workflow for building 3D models in Metashape was followed using the parameter values summarised in Table S3: Alignment, Camera Optimisation, Dense Cloud construction, 3D mesh creation and Mesh texturing. Resulting models had >93% photo alignment on average and an average (± SD) resolution (distance between mesh vertices) of 1.1 (0.4) mm and average (± SD) scaling error of 0.36 (0.38) mm.

3D model comparisons. We used Geomagic Control (v 2015, 3D Systems) to clean and align successive 3D colony models, and extract size metrics. Each model was exported from Agisoft Metashape as an .obj file type and imported into Geomagic. The MeshDoctor tool was run to detect and remove any photogrammetric artefacts (features selected with the tool included non-manifold edges, self-intersections, highly creased edges, small components, small tunnels and small holes). All cleaned mesh models for a given colony were then imported into Geomagic simultaneously and aligned. 2017 was the base case for alignment except where this was not available (a few colonies were first imaged in 2018 or, in some instances, the 2017 model failed to build). Alignment was conducted in two stages. Initial alignment between a pair of models was done using the N-Point tool in Geomagic Control where at least three points common to both models were identified for a course-level alignment. Next, the Best-fit tool was used but allowing only fine adjustments and 10,000 iterations to refine the initial course-level fitting. Once aligned, all models were trimmed simultaneously to give them a common base. The surface area of each model was obtained using the Analysis Compute tool. The base of each model was then closed using the Fill Single tool (with flat plane) and the Analysis Compute tool used to derive the overall volume. A copy of the models pre-base filling were also trimmed to leave just the live area of each colony and the Analysis Compute tool used to obtain the surface area of this live component only.

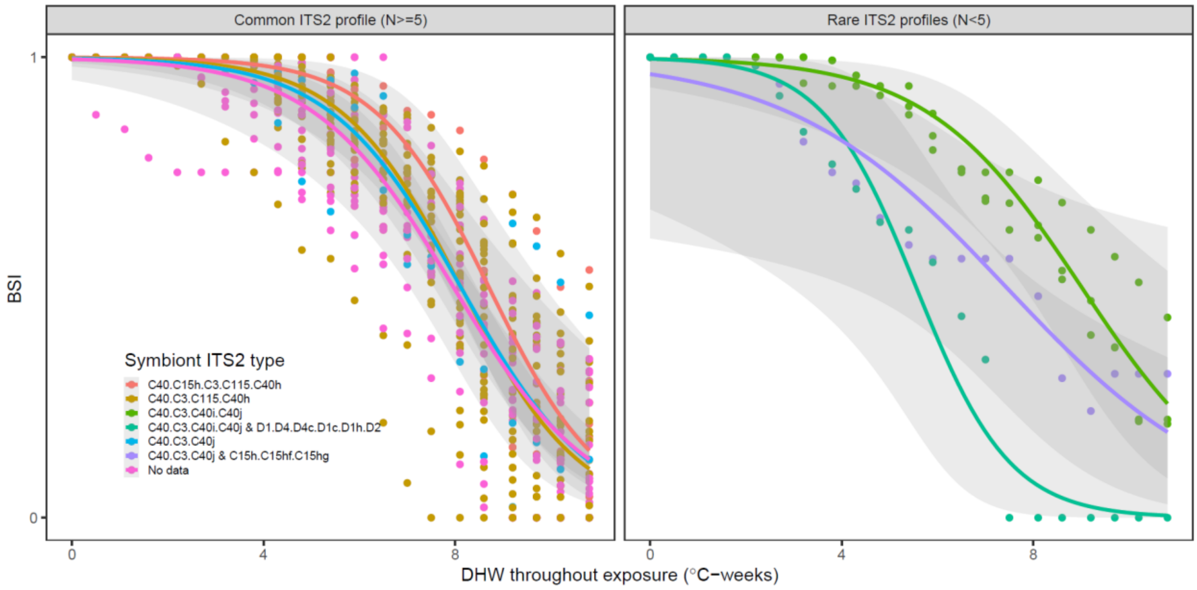

Fig. S1. Effect of heat stress (DHW) on bleaching survival index (BSI) for each Symbiodinaceae ITS2 type profile mixture present in the different colonies.

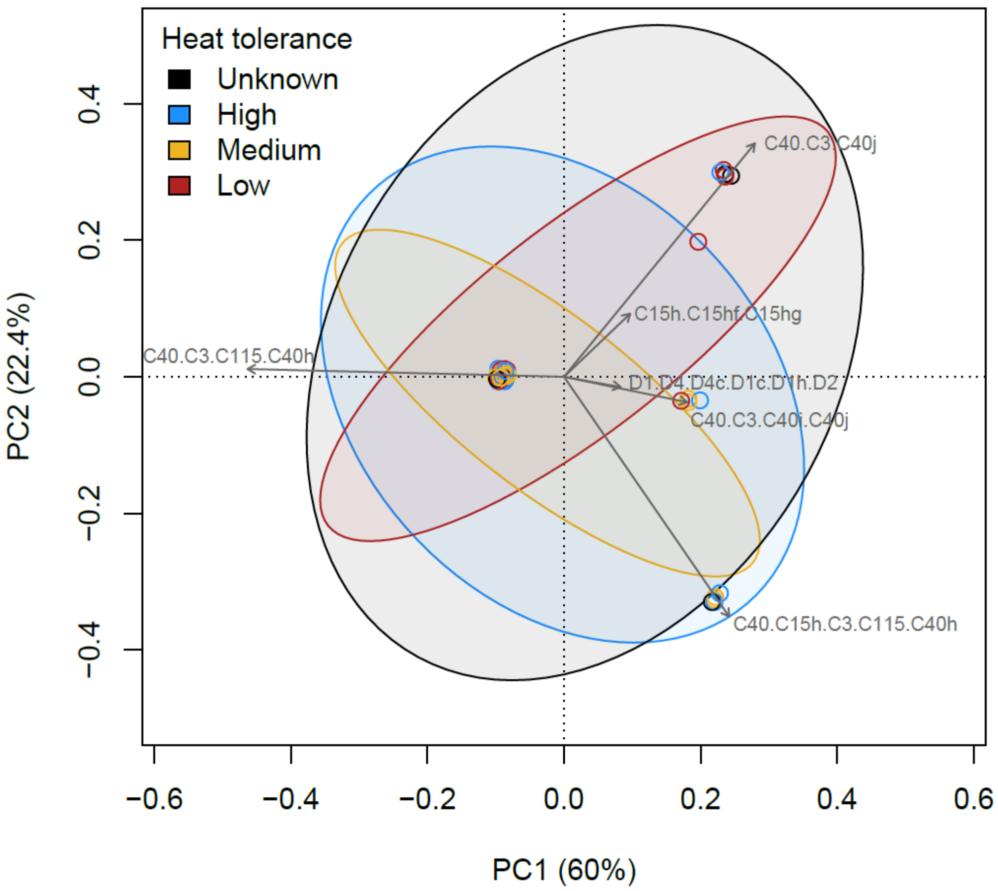

Fig. S2. Principal component analysis of the Symbiodinaceae community associated with sampled corals grouped according to their heat tolerance, which was transformed to a categorical variable based on average BSI (High ≥ 0.8 > Medium ≥ 0.7 > Low), showing the proportion of variability explained by the first two principal components (brackets), and 95% confidence interval of the standard deviation shown as ellipsoids.

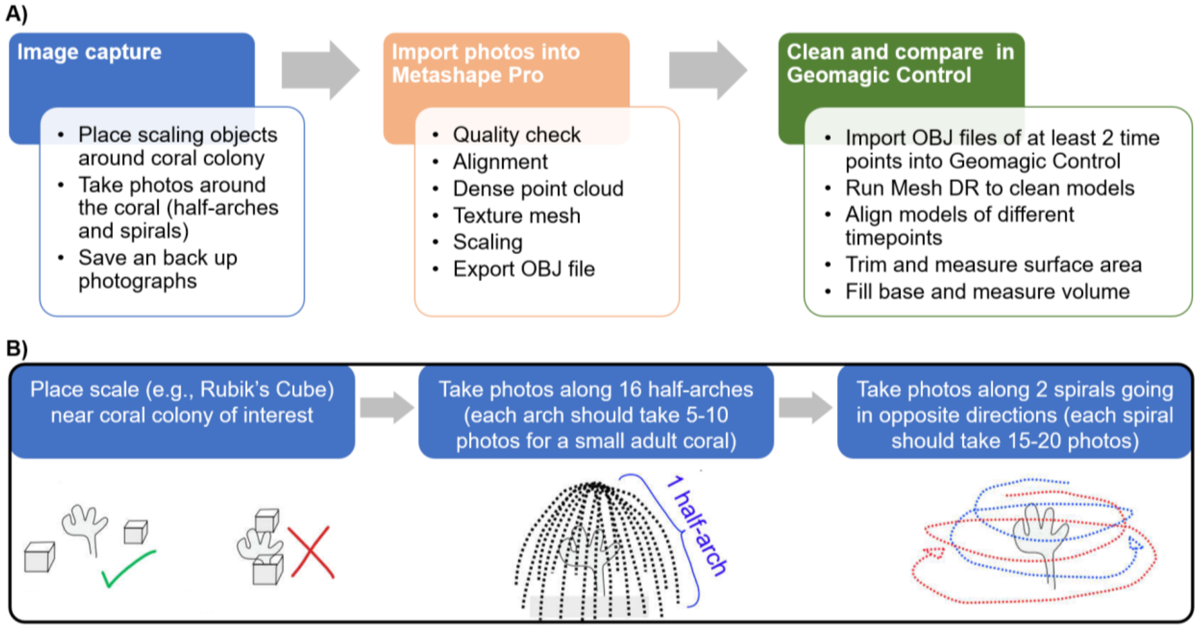

Fig. S3. Overview of photogrammetry methodology. (A) The main three steps in photogrammetry surveys for coral colony growth, from image acquisition to 3D model building, and finally interannual model comparisons. (B) Further details on image acquisition.

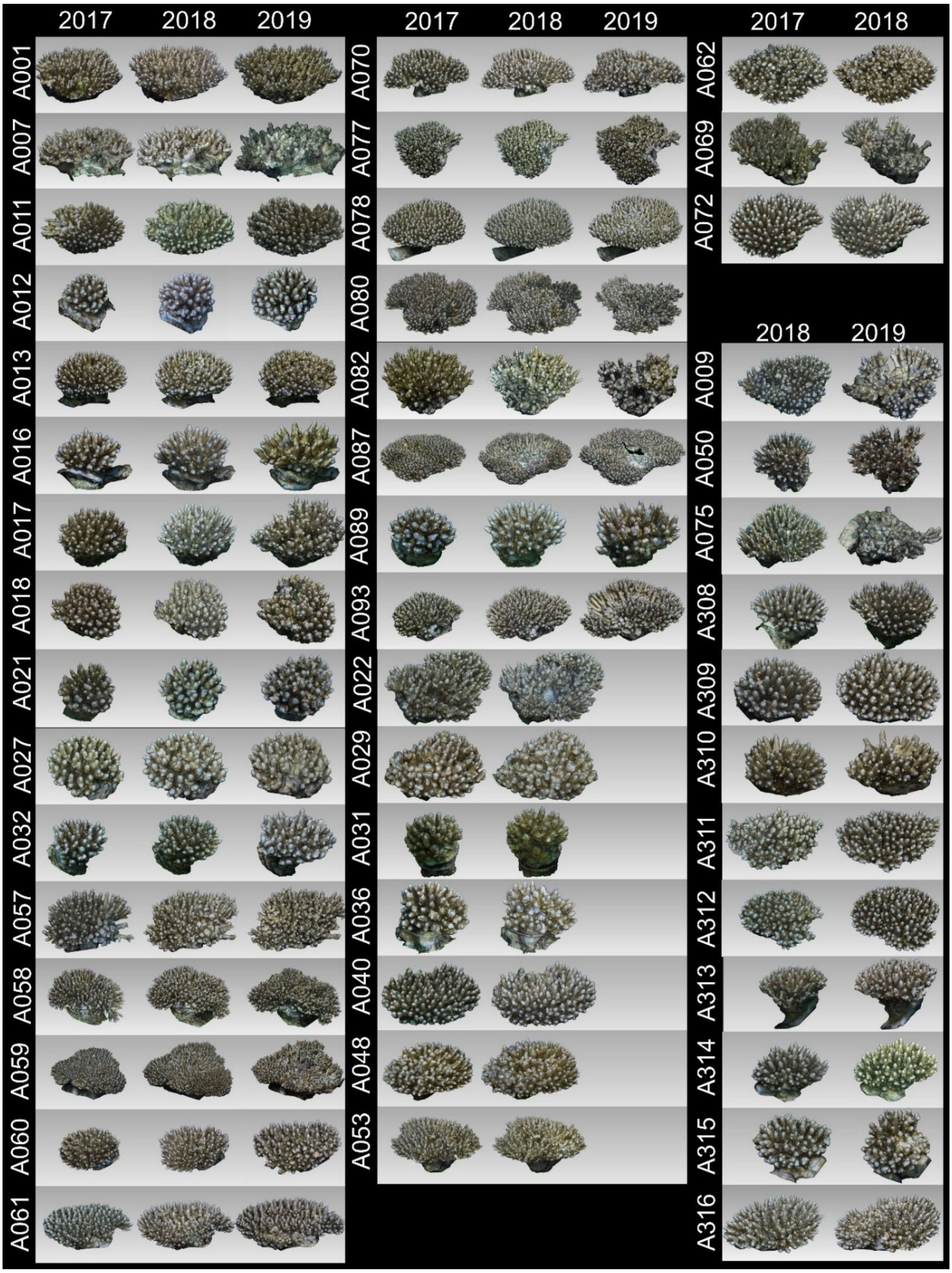

Fig. S4. Profiles of each *Acropora digitifera* 3D photogrammetry model (y-axis) across the available years for that colony (x-axis). 3D model profile shots were taken from the same direction each year and scaled relative to that colony. Thus, size comparisons across years can be made for one colony visually, but size comparisons among colonies are not valid in this figure.

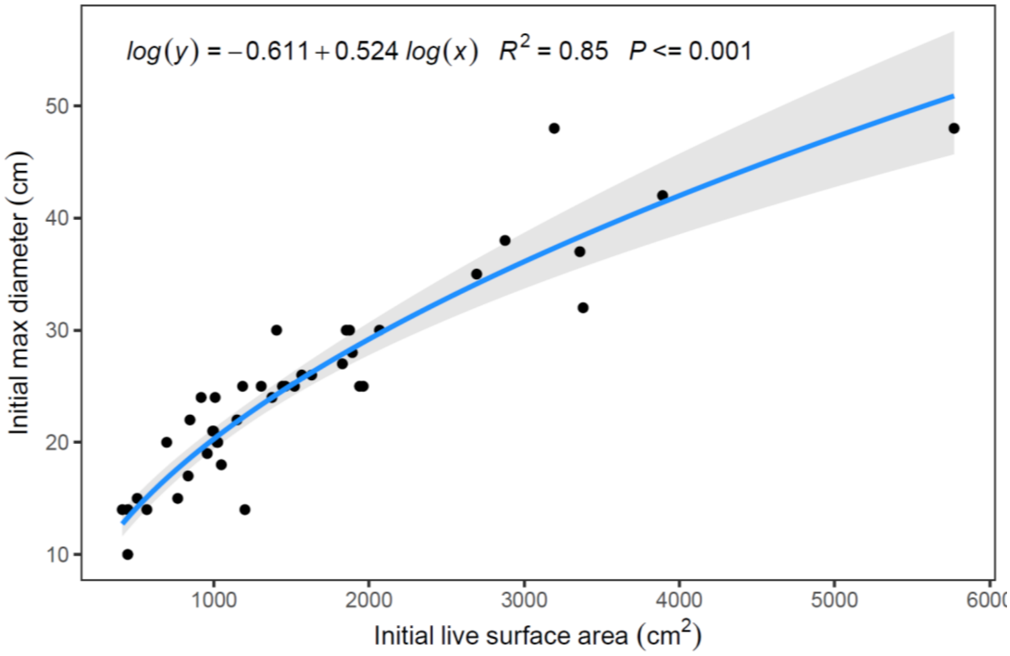

Fig. S5. Relationship between initial live surface area from 3D photogrammetry models and initial maximum diameter measured in the field using a measuring tape. This relationship was used to predict initial maximum diameter for colonies lacking empirical measurements used in Fig. S6.

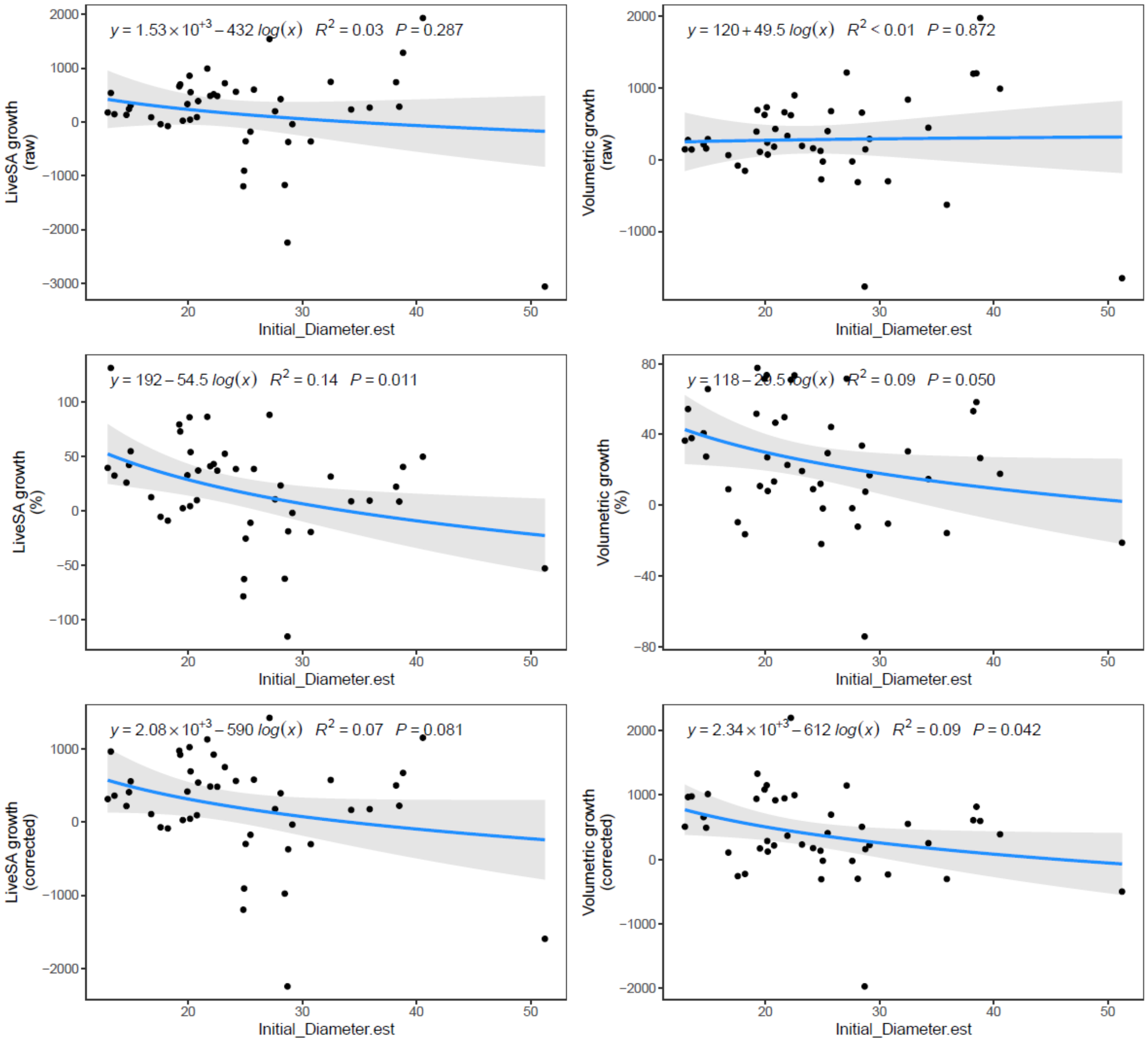

Fig. S6. Size dependencies for live surface area growth metrics (LiveSA, left) and volumetric growth metrics (right) are shown using raw values (top), percentages (middle), and size-corrected values (bottom). The amount of variability in photogrammetry-derived growth metrics (response variables) that can be explained by independently estimated colony size, or initial maximum diameter (predictor variable), is shown with regression equations (based on log-transformed diameter), R^2 values, and P values.

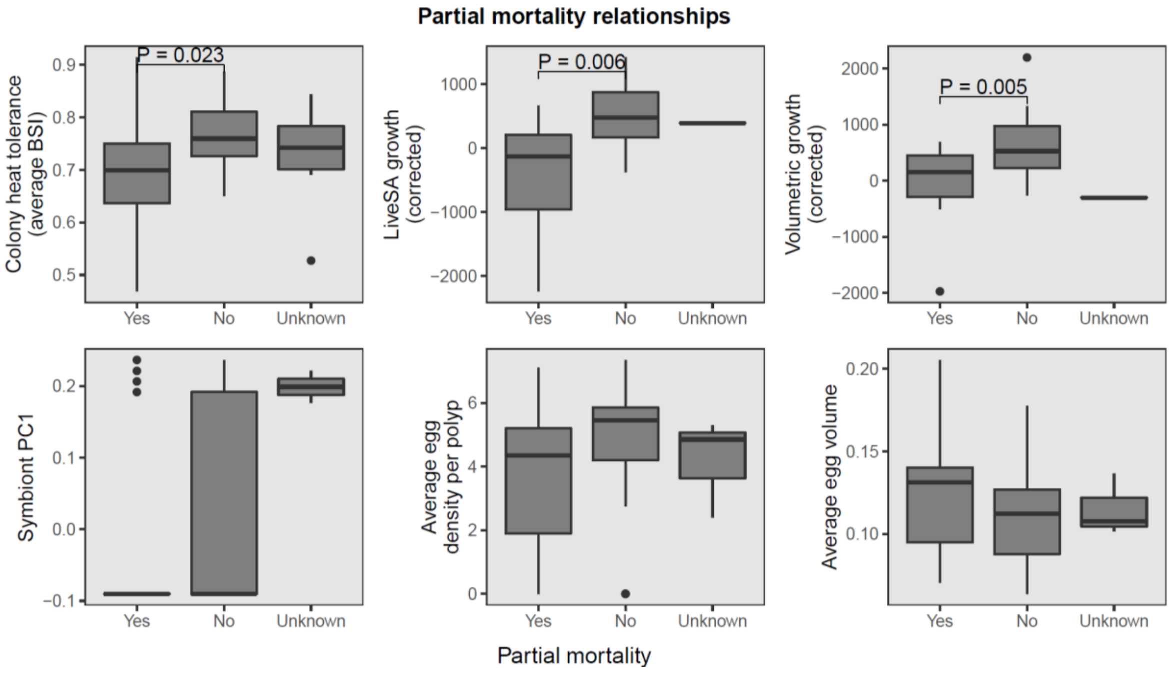

Fig. S7. Relationships between partial mortality and all other traits. Significant pairwise comparisons among partial mortality categories are shown with brackets and P values and are based on ANOVAs linked to pairwise Tukey tests. Wilcoxon rank sum tests with pairwise Bonferroni corrections were used when there was heterogeneity of variance among groups, or the groups were not normally distributed.

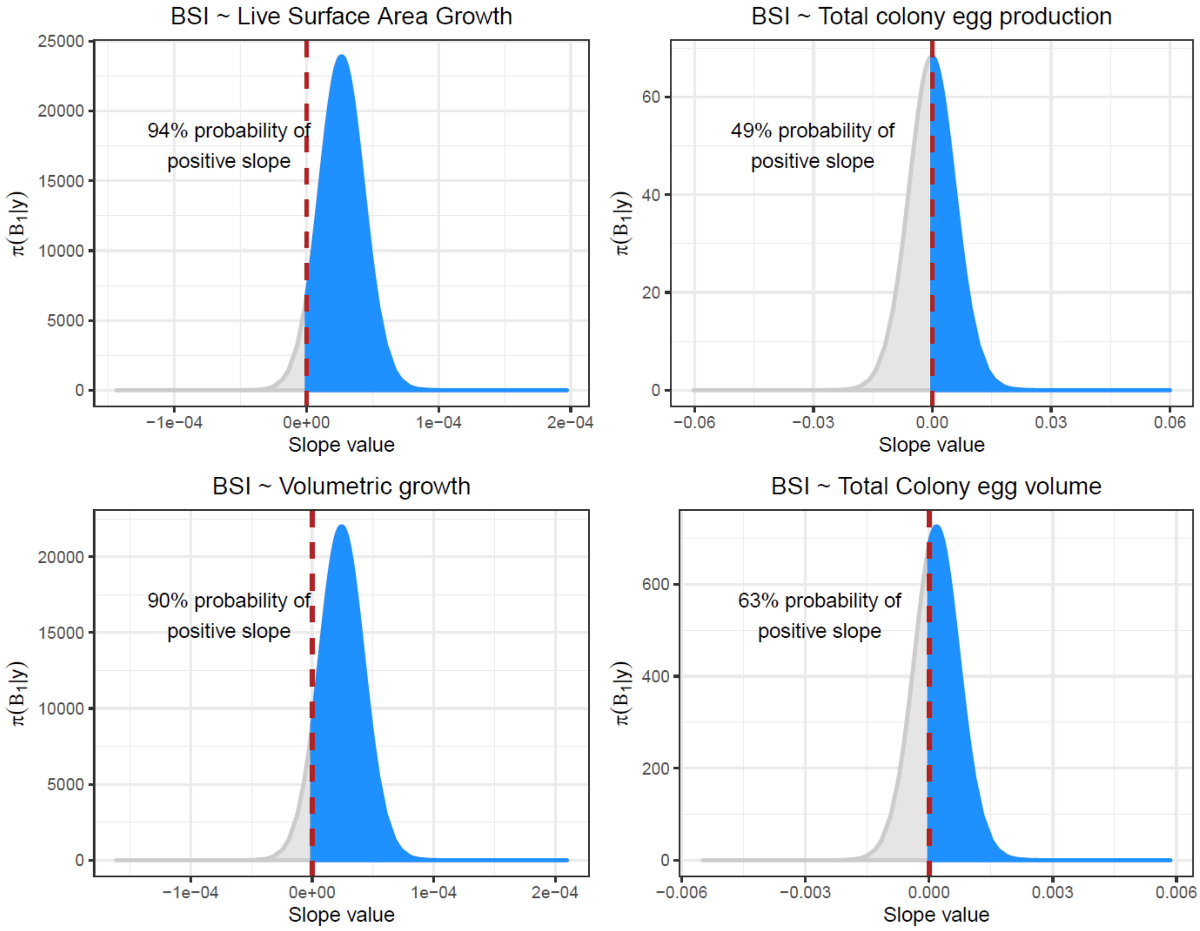

Fig. S8. Posterior distribution estimates of β_0 given the data (*i.e.*, the slope of linear regressions in the form: average BSI ~ Trait). Trade-offs between fitness traits and heat tolerance would appear as negative slopes, whereas co-benefits among traits would appear as positive slopes. Therefore, the probability of a positive slope (or a co-benefit) is shown as the computation of the percentage area under the posterior distribution that is > 0 (red dashed line, no relationship).

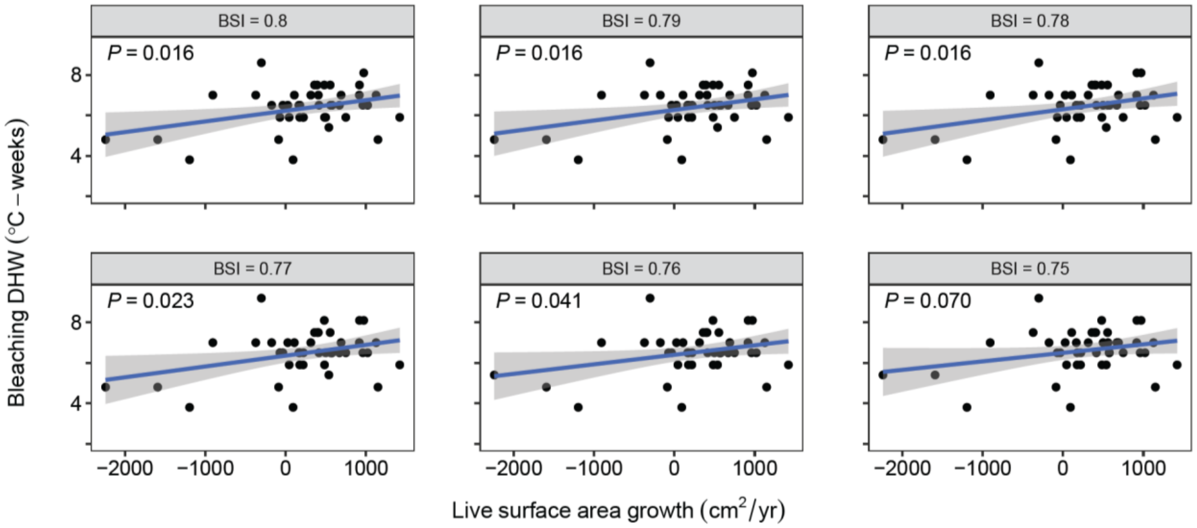

Fig. S9. Delays in the onset of bleaching depending on colony growth, showing corrected live surface area growth (x-axis), and the bleaching dosage of DHW calculated as the DHW at which BSI reduces under a bleaching cut-off. We have shown these results for a bleaching BSI cut-off ranging from 0.8 to 0.75, which could occur with all four replicate fragments partially bleached (BSI=0.75) and other variations of fragment health status categories.

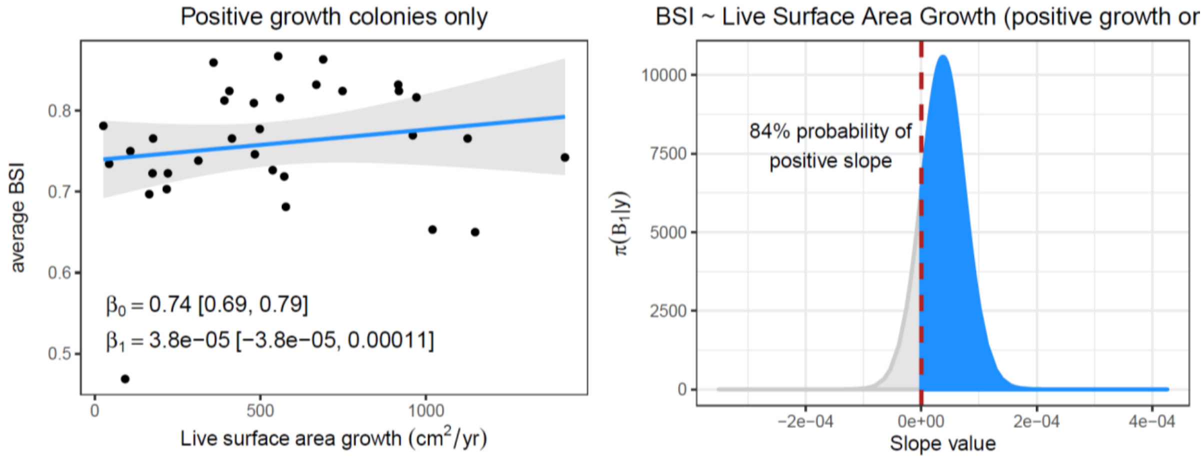

Fig. S10. Associations (left) between colony heat tolerance (average BSI through time) and corrected live surface area growth for positive growth colonies only, showing the median and 95% credible interval of intercept and slope parameters of linear regressions. The posterior distribution of β_1 given the data is shown on the right. Co-benefits among traits would appear as positive slopes. Therefore, the probability of a positive slope is shown as the percentage area under the posterior distribution that is > 0 (red dashed line, no relationship)..

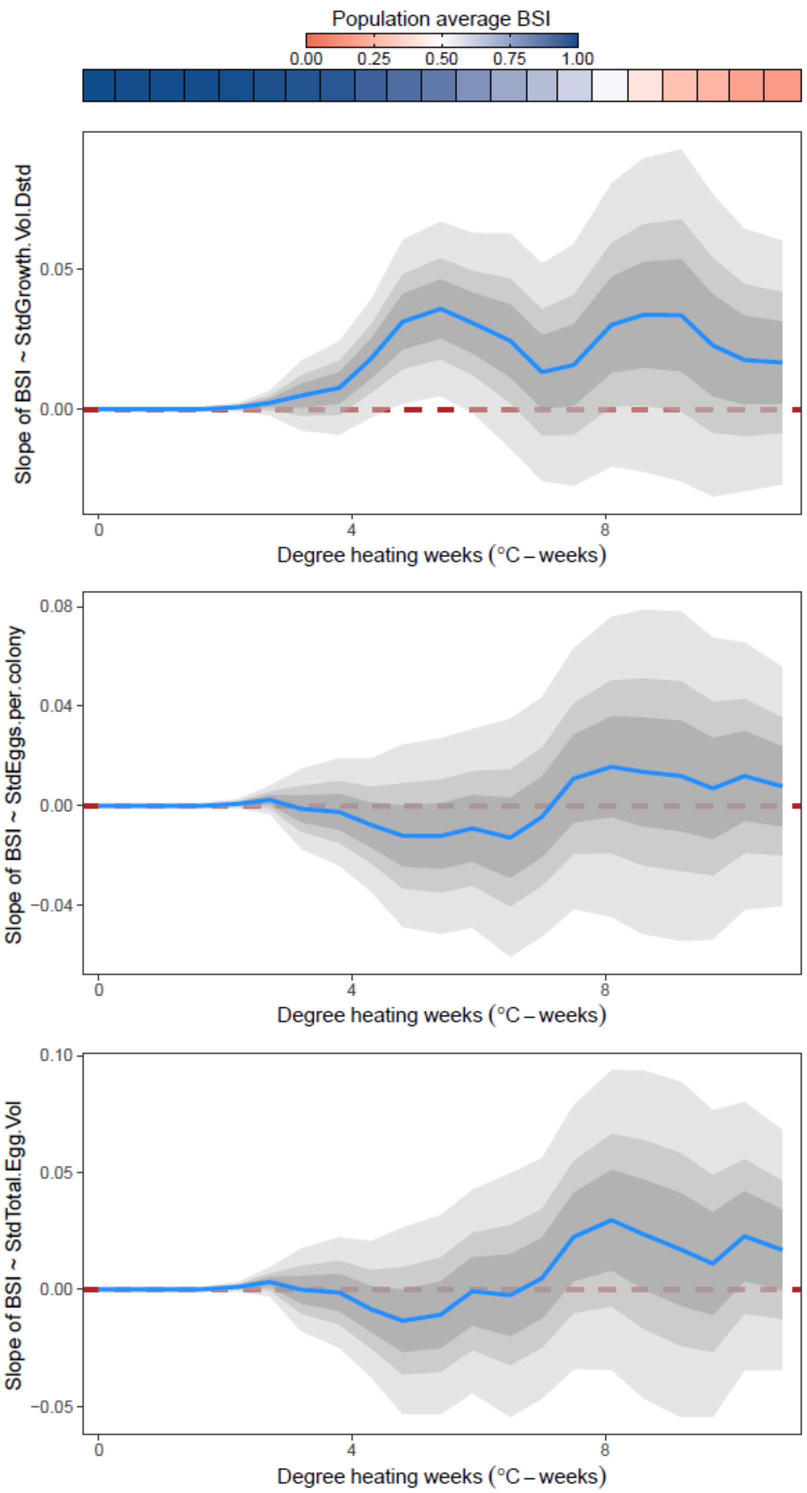

Fig. S11. BSI-trait relationships shown as slope estimates (posterior distribution mean) and credible intervals of the posterior distribution (50, 75, and 95% as dark, medium, and light grey, respectively) throughout the heat stress exposure (x-axis). The traits shown are volumetric growth (top), total egg number per colony (middle), and total egg volume per colony (bottom).

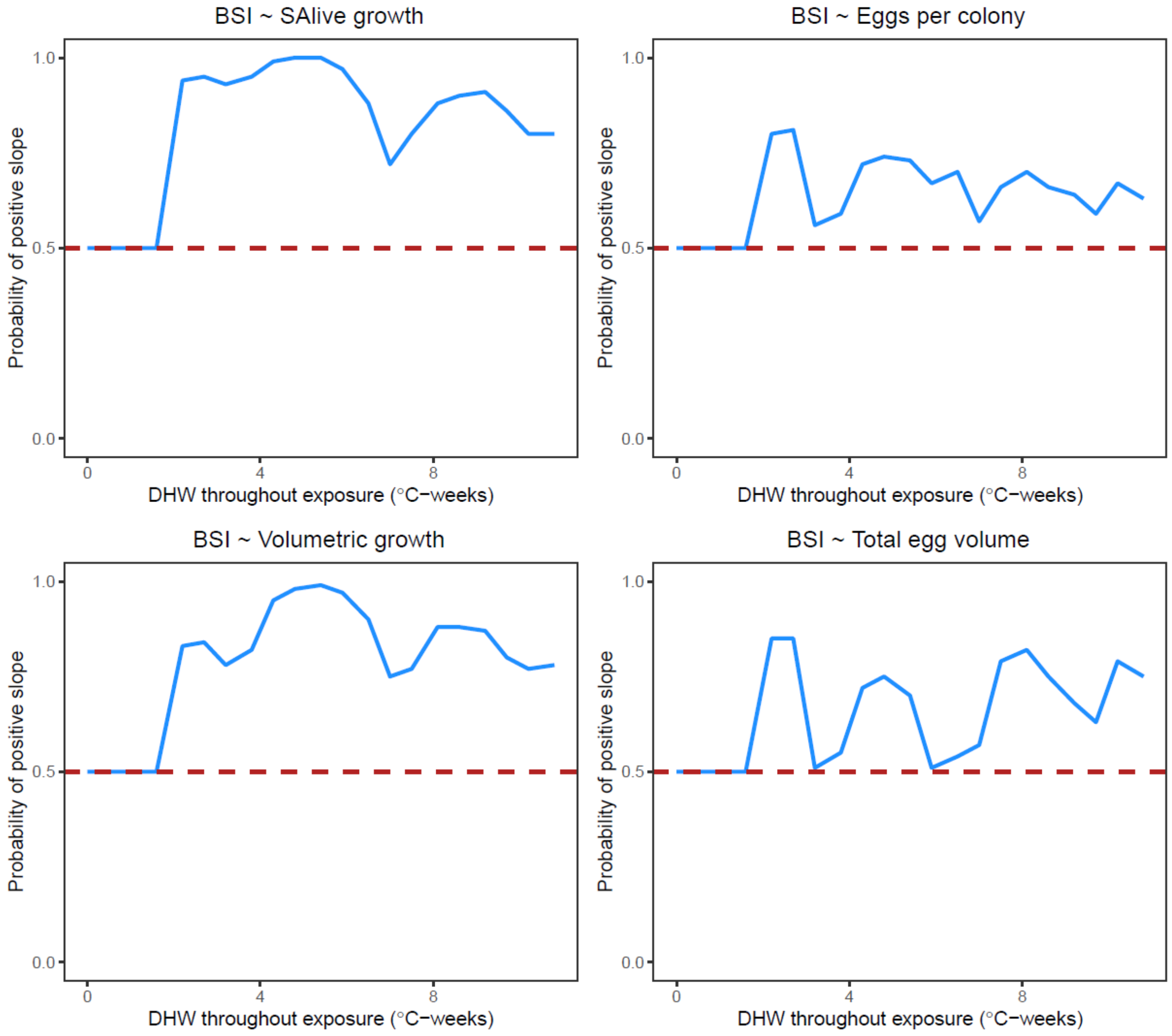

Fig. S12. The probability of a positive slope (or a co-benefit) for BSI~trait relationships throughout the DHW exposure and throughout bleaching and mortality responses, based on the proportion of area of the β_0 posterior distribution > 0. Trade-offs between fitness traits and heat tolerance would appear as negative slopes, whereas co-benefits among traits would appear as positive slopes.

Table S1. Table of metadata associated to heat stress experiment following [4].

Metadata	Conditions or methods
Coral collection	Latitude and longitude N 07°17' 29.3"; E 134°31' 8.0"
	Collection depth: between 2 and 3 m depth
	Collection dates: Between July 27th and August 1st 2018
	Coral species: *Acropora digitifera*
	Coral morphology: corymbose
	Symbiodinaceae for all colonies: ITS2 marker
	Acclimation post collection prior to experiment: between 7 and 12 days
Experimental design	Name of location: Palau International Coral Reef Center, Palau
	Bleaching stress temperature period: from August 8th until 12th September 2018
	System type: outdoor flow through system
	No. tanks per treatment: 4 temperature stress tanks and 2 procedural control tanks
	No. coral genets (colonies) per treatment: 66
	No. coral genets (colonies) per tank within treatments: 66
Experimental temperature conditions	Heat stress temperature above MMM per treatment: approximately + 3.5 °C above the local climatological baseline, MMM_{adj} (see baseline temperature description below)
	Control temperature: 29.28 ± 0.71 °C
	Baseline temperature: CoralTemp MMM was 29.2 °C but adjusted to 29.4 °C based on the relationship between satellite sea surface temperatures and in situ temperatures. Stress baseline for accumulating DHW was set to MMM_{adj} + 1 °C
	Temperature ramp up rate: Compared to the MMM_{adj}, temperatures in heated tanks were adjusted by (A) + 1 ºC on day 1 to ~30.5 ºC; (B) + 0.5 ºC on days 12, 18, and 26 to a temperature of ~32 ºC; and (C) +1 ºC on day 30 to 33 ºC
	Duration at heat stress temperature: 35 days
	Temperature modulation: diurnal
Other experimental conditions	Light conditions: 400 µmol photons m^{-2} s^{-1}
	Light cycle: 12 h:12 h diurnal cycle
	Water flow velocity: 7.2 L/hour
	Tank turnover: 2 times per day
	Sea water filtration: 50µm filtered
	Sea water source: natural directly from the reef
	Salinity: 35‰
	Feeding: natural feeding from plankton in the filtered seawater

Table S2. Pairwise Tukey comparisons of bleaching survival index (BSI) among mixtures of Symbiodinaceae ITS2 type profiles present in the different colonies. Based on the GLMM in the form: BSI ~ DHW * Symbiont.Group + (1|Colony_ID). DF = 1329. Comparisons among symbiont groups are shown for an average DHW of 5.4 ºC-weeks. The interaction term of the model was not significant.

**Contrast**	**Odds ratio**	**SE**	**t ratio**	**P value**
C40.C15h.C3.C115.C40h / C40.C3.C115.C40h	1.85	1.34	0.85	0.98
C40.C15h.C3.C115.C40h / C40.C3.C40i.C40j	1.74	1.61	0.60	1.00
C40.C15h.C3.C115.C40h / C40.C3.C40i.C40j & D1.D4.D4c.D1c.D1h.D2	13.99	14.44	2.56	0.14
C40.C15h.C3.C115.C40h / C40.C3.C40j	2.21	2.02	0.87	0.98
C40.C15h.C3.C115.C40h / C40.C3.C40j & C15h.C15hf.C15hg	6.73	6.24	2.06	0.38
C40.C15h.C3.C115.C40h / No data	2.83	2.06	1.43	0.78
C40.C3.C115.C40h / C40.C3.C40i.C40j	0.94	0.60	-0.10	1.00
C40.C3.C115.C40h / C40.C3.C40i.C40j & D1.D4.D4c.D1c.D1h.D2	7.55	5.94	2.57	0.14
C40.C3.C115.C40h / C40.C3.C40j	1.19	0.74	0.29	1.00
C40.C3.C115.C40h / C40.C3.C40j & C15h.C15hf.C15hg	3.64	2.34	2.01	0.41
C40.C3.C115.C40h / No data	1.53	0.43	1.51	0.74
C40.C3.C40i.C40j / C40.C3.C40i.C40j & D1.D4.D4c.D1c.D1h.D2	8.04	7.83	2.14	0.33
C40.C3.C40i.C40j / C40.C3.C40j	1.27	1.08	0.28	1.00
C40.C3.C40i.C40j / C40.C3.C40j & C15h.C15hf.C15hg	3.87	3.33	1.57	0.70
C40.C3.C40i.C40j / No data	1.63	1.04	0.76	0.99
C40.C3.C40i.C40j & D1.D4.D4c.D1c.D1h.D2 / C40.C3.C40j	0.16	0.15	-1.92	0.47
C40.C3.C40i.C40j & D1.D4.D4c.D1c.D1h.D2 / C40.C3.C40j & C15h.C15hf.C15hg	0.48	0.47	-0.75	0.99
C40.C3.C40i.C40j & D1.D4.D4c.D1c.D1h.D2 / No data	0.20	0.16	-2.03	0.40
C40.C3.C40j / C40.C3.C40j & C15h.C15hf.C15hg	3.04	2.58	1.31	0.85
C40.C3.C40j / No data	1.28	0.80	0.40	1.00
C40.C3.C40j & C15h.C15hf.C15hg / No data	0.42	0.27	-1.35	0.83

Table S3. Overview of the parameter values used for the standard 3D model building workflow using Metashape.

Processes	Parameters
Alignment	High accuracy, pair and reference preselection disabled, key point limit 40,000, tie point limit 4000, features not constrained by mask, no adaptive camera model fitting
Sparse cloud	All optimisation properties 'yes' except fit b1, b2, k4, p3, p4, adaptive camera model fitting
Dense point cloud	High quality *, mild depth filtering, do not reuse depth maps, calculate point colours
Mesh	Arbitrary surface type, source data-dense cloud, face count high, interpolation enabled, all point classes, don't reuse depth maps
Texture	Generic mapping mode, texture from all cameras, mosaic blending mode, texture size 8192, texture count 1, hole filling
Scaling	2 x independent horizontal and vertical features per colony

Supplementary References

1. Voolstra, C. R. *et al.* Standardized short-term acute heat stress assays resolve historical differences in coral thermotolerance across microhabitat reef sites. *Glob. Chang. Biol.* **26**, 4328–4343 (2020).

2. Hume, B. C. C. *et al.* SymPortal: A novel analytical framework and platform for coral algal symbiont next-generation sequencing ITS2 profiling. *Mol. Ecol. Resour.* **19**, 1063–1080 (2019).

3. Ferrari, R. *et al.* 3D photogrammetry quantifies growth and external erosion of individual coral colonies and skeletons. *Sci. Rep.* **7**, 1–9 (2017).

4. Grottoli, A. G. *et al.* Increasing comparability among coral bleaching experiments. *Ecol. Appl.* **31**, 1–17 (2021).
