# Supplementary material for: No apparent trade-offs associated with heat tolerance in a reef-building coral
Source: Commun Biol. 2023 Apr 12;6:400. doi: 10.1038/s42003-023-04758-6 (PMC10097654; doi:10.1038/s42003-023-04758-6)
Supplement: Supplementary file 3 — Reporting Summary [file 42003_2023_4758_MOESM3_ESM.pdf]

Corresponding author(s): Liam Lachs

Last updated by author(s): Mar 13, 2023

Reporting Summary

Nature Portfolio wishes to improve the reproducibility of the work that we publish. This form provides structure for consistency and transparency in reporting. For further information on Nature Portfolio policies, see our Editorial Policies and the Editorial Policy Checklist.

Statistics

For all statistical analyses, confirm that the following items are present in the figure legend, table legend, main text, or Methods section.

n/a	Confirmed	
☐	☒	The exact sample size (*n*) for each experimental group/condition, given as a discrete number and unit of measurement
☐	☒	A statement on whether measurements were taken from distinct samples or whether the same sample was measured repeatedly
☐	☒	The statistical test(s) used AND whether they are one- or two-sided<br>*Only common tests should be described solely by name; describe more complex techniques in the Methods section.*
☐	☒	A description of all covariates tested
☐	☒	A description of any assumptions or corrections, such as tests of normality and adjustment for multiple comparisons
☐	☒	A full description of the statistical parameters including central tendency (e.g. means) or other basic estimates (e.g. regression coefficient) AND variation (e.g. standard deviation) or associated estimates of uncertainty (e.g. confidence intervals)
☐	☒	For null hypothesis testing, the test statistic (e.g. *F*, *t*, *r*) with confidence intervals, effect sizes, degrees of freedom and *P* value noted<br>*Give P values as exact values whenever suitable.*
☒	☐	For Bayesian analysis, information on the choice of priors and Markov chain Monte Carlo settings
☒	☐	For hierarchical and complex designs, identification of the appropriate level for tests and full reporting of outcomes
☒	☐	Estimates of effect sizes (e.g. Cohen's *d*, Pearson's *r*), indicating how they were calculated

Our web collection on statistics for biologists contains articles on many of the points above.

Software and code

Policy information about availability of computer code

Data collection	no software was used to collect data
Data analysis	R version 4.0.2; Agisoft Metashape Professional version 1.5; 3D Systems Geomagic Control version 2015

For manuscripts utilizing custom algorithms or software that are central to the research but not yet described in published literature, software must be made available to editors and reviewers. We strongly encourage code deposition in a community repository (e.g. GitHub). See the Nature Portfolio guidelines for submitting code & software for further information.

Data

Policy information about availability of data

All manuscripts must include a data availability statement. This statement should provide the following information, where applicable:

- Accession codes, unique identifiers, or web links for publicly available datasets
- A description of any restrictions on data availability
- For clinical datasets or third party data, please ensure that the statement adheres to our policy

All original data and R code produced in this study are publicly available on Figshare at 10.25405/data.ncl.20411589 (until publication, the repository is link-access only and can be found at https://figshare.com/s/f1016b972bd2f159faa2). ITS2 sequences have been archived publicly at NCBI under BioProject 864615 and processed symbiont community composition can be explored publicly at https://symportal.org. Any additional information required to reanalyse the data reported in this paper is available from the lead contact upon request.

Human research participants

Policy information about studies involving human research participants and Sex and Gender in Research.

Reporting on sex and gender	NA
Population characteristics	NA
Recruitment	NA
Ethics oversight	NA

Note that full information on the approval of the study protocol must also be provided in the manuscript.

Field-specific reporting

Please select the one below that is the best fit for your research. If you are not sure, read the appropriate sections before making your selection.

☐ Life sciences ☐ Behavioural & social sciences ☒ Ecological, evolutionary & environmental sciences

For a reference copy of the document with all sections, see nature.com/documents/nr-reporting-summary-flat.pdf

Ecological, evolutionary & environmental sciences study design

All studies must disclose on these points even when the disclosure is negative.

Study description	A field study on 70 individual reef-building Acropora digitifera corals to test for trade-offs between coral heat tolerance and three other traits: growth, fecundity and symbiont community composition. Each trait was measured for each colony where possible (some missing data points for certain colonies due to logistical constraints).
Research sample	A group of tagged Acropora digitifera, reef-building corals, on a single reef. One reef was used as the objective was to determine within-population variability in traits (as natural selection for corals occurs over local scales), and limit the influence of environmental factors on study outcomes. A. digitifera is a hermaphroditic broadcast spawning coral. Mid to large sized colonies were chosen to limit the influence of size on study outcomes, as well as to be able to have sufficient size to remove some fragments without causing damage to the colony.
Sampling strategy	To have enough statistical power to detect weak trends in noisy ecological data, large sample sizes are necessary. However, due to space and logistical limits, a maximum of 70 colonies could be used for the heat stress assay, at a replication of 4 heat stressed branches per colony to account for iwithin-colony variation (which was much smaller than between-colony variation). This set the max sample size for other trait measurements which was then limited other factors including colonies being found on the reef during underwater field surveys.
Data collection	Heat tolerance: building a tank system, calibrating thermometers, collecting coral fragments, running assay - AH, HMM, LL, AJE, and JRG<br>Symbiont ID: Collecting samples, DNA extraction, PCR, sending for sequencing and bioinformatics - AH, EB, ES, JRG, LL, and JCB<br>Size and growth: Doing 3D photogrammetry surveys, building 3D models, cleaning 3D models, measuring size and growth metrics - DRP, LL, BS, WFF, and RF.<br>Fecundity: Collecting fragments, decalcifying and preserving samples, dissecting polyps and image analysis - LL, AH, HMM, ES, and JRG
Timing and spatial scale	Spatial scale: within a single population on Maschercher reef in Palau, Micronesia<br>Sampling time period: November 2017 to February 2019<br>Sampling time points: heat tolerance (August 2018), symbiont ID (March 2018 and two missed colonies in September 2018), size and growth (November 2017, May 2018, and February 2019 to achieve two growth comparisons), fecundity (March 2018 and March 2019 for colonies missed in 2018).
Data exclusions	No data were excluded for analyses
Reproducibility	The heat stress assay and symbiont ID have been repeated on different sets of colonies in different years, showing similar heat tolerances for this species in each. The 3D photogrammetry surveys and fecundity surveys were ecological field survey type experiments and given the labour intensive effort to generate these datasets, it was deemed inappropriate to reproduce this work for this study.
Randomization	Tagged coral colonies were located randomly across the home reef. Samples from these colonies were always taken from the center of the colony to avoid intracolonial biases in phenotypic measurements. In the marine heatwave emulation experiment, fragments (6 per colony) were dispersed in random locations among the four heat stress tanks (4 fragments/colony) and two procedural control tanks (2 fragments/colony).

Blinding	Particularly for the marine heatwave assay, each colony fragment was given a random number and randomly dispersed in the tanks thus not allowing the observer who was assigning health status scores daily to know the colony from which the fragments were collected.

Did the study involve field work? ☒ Yes ☐ No

Field work, collection and transport

Field conditions	No major disturbances occurred during the 2-year study (marine heatwaves or typhoons)
Location	Maschercher reef, Palau. N 07°17' 29.3''; E 134°31' 8.0''
Access & import/export	All efforts were made to collect and export samples in compliance with local, national and international laws. Less than 10% of the volume of each colony was collected from tagged colonies for experimental work. Both national and state permits were obtained prior to any work commencing and all work was done with full collaboration of the Palau International Coral Reef Center. This work was conducted using Koror State permits (018, 032, 034, 037) and Palau National permits (RE-18-13, RE-19-08), and CITES export permits (permit number PW19-111).
Disturbance	The aim of this study was not to impact on the population of corals. Only several fragments were sampled from each coral colony at less than 10% of the colony volume. All other methods were non-invasive. The survival of colonies on the reef after 3 years was comparable to natural survival rates (~11% per year)

Reporting for specific materials, systems and methods

We require information from authors about some types of materials, experimental systems and methods used in many studies. Here, indicate whether each material, system or method listed is relevant to your study. If you are not sure if a list item applies to your research, read the appropriate section before selecting a response.

Materials & experimental systems

n/a	Involved in the study	
☒	☐	Antibodies
☒	☐	Eukaryotic cell lines
☒	☐	Palaeontology and archaeology
☐	☒	Animals and other organisms
☒	☐	Clinical data
☒	☐	Dual use research of concern

Methods

n/a	Involved in the study	
☒	☐	ChIP-seq
☒	☐	Flow cytometry
☒	☐	MRI-based neuroimaging

Animals and other research organisms

Policy information about studies involving animals; ARRIVE guidelines recommended for reporting animal research, and Sex and Gender in Research

Laboratory animals	This study did not involve laboratory animals
Wild animals	This study involved tagged corals on the reef and monitoring them for various traits from 2017 to 2019.
Reporting on sex	The species we were working with (Acropora digitifera) is hermaphroditic.
Field-collected samples	Heat tolerance assay samples: After fragments were collected and given a 7–10-day acclimatisation period, temperature was increased gradually over the time course of the experiment (35 days), reaching a final bleaching-level temperature of approximately 33 ºC, or 3.5 ºC above the local climatological baseline (Fig. 1A, Table S1). The use of flow-through tank systems allowed an element of natural diel temperature variability in all tanks (4 heat stress and 2 procedural control tanks), while aquarium lights provided light conditions close to those measured on the reef.<br>Symbiont ID samples: The composition of the symbiont community was identified from one tissue scraping (<1 cm) per colony, stored in ethanol. DNA was extracted using the Qiagen DNeasy blood and tissue kit with overnight proteinase K digestion. Polymerase chain reaction (PCR) was used to amplify DNA extracted from coral tissue and then sent for ITS2 sequencing.<br>Fecundity samples: Two fragments from each tagged coral colony in the reef were removed prior to spawning in March 2018 or 2019, decalcified using 10% hydrochloric acid and stored in ethanol.
Ethics oversight	The research presented here adhered to the ethical standards consistent with LL's institutional and internal review board policies and received their approval.

Note that full information on the approval of the study protocol must also be provided in the manuscript.
